# Care for hospitalized patients with unhealthy alcohol use: a narrative review

**DOI:** 10.1186/1940-0640-8-11

**Published:** 2013-06-05

**Authors:** Regina Makdissi, Scott H Stewart

**Affiliations:** 1 Division of General Internal Medicine, University at Buffalo State University of New York, Buffalo, NY, USA

**Keywords:** Alcohol drinking, Alcoholism, Hospitalization, Patient discharge

## Abstract

There is increasing emphasis on screening, brief intervention, and referral to treatment (SBIRT) for unhealthy alcohol use in the general hospital, as highlighted by new Joint Commission recommendations on SBIRT. However, the evidence supporting this approach is not as robust relative to primary care settings. This review is targeted to hospital-based clinicians and administrators who are responsible for generally ensuring the provision of high quality care to patients presenting with a myriad of conditions, one of which is unhealthy alcohol use. The review summarizes the major issues involved in caring for patients with unhealthy alcohol use in the general hospital setting, including prevalence, detection, assessment of severity, reduction in drinking with brief intervention, common acute management scenarios for heavy drinkers, and discharge planning. The review concludes with consideration of Joint Commission recommendations on SBIRT for unhealthy alcohol use, integration of these recommendations into hospital work flows, and directions for future research.

## Background

Alcohol-related problems constitute a tremendous economic and health cost in many countries throughout the world [1,2]. Screening, brief intervention, and referral to treatment (SBIRT) has become one of the major tools used to combat these problems, and is widely recommended for use in primary care by governments and expert panels [3,4]. Recently, the Joint Commission on Accreditation for Health Care Organizations (JCAHO), the major accrediting body for hospitals in the US, has advanced SBIRT as a quality indicator for general hospital care [5]. There is no doubt that alcohol problems are a critical issue in hospital care, but there are concerns that the evidence base does not yet justify widespread SBIRT in this setting [6]. This review will consider the nature of unhealthy alcohol use in the general hospital, review means for detecting unhealthy use and categorizing severity, consider the evidence on the effectiveness of SBIRT in the hospital setting, briefly review acute treatment issues, and discuss factors affecting the implementation of SBIRT into general hospital workflows. The goal is to provide an overview of the field that will aid hospital-based clinicians and administrators in their consideration of policies and procedures that will yield the highest quality care for their patients. In this regard, the impact of detecting and treating unhealthy alcohol use is one of many important health care issues that must be evaluated.

### Unhealthy alcohol use in the hospital

Unhealthy alcohol use is an umbrella term encompassing any pattern of alcohol use that increases risk for or causes physical problems [7]. At the lower end of severity, unhealthy use is often operationalized as drinking in excess of health-related guidelines. In the US, the National Institute on Alcohol Abuse and Alcoholism defines this as more than one drink daily, more than 7 drinks weekly, or exceeding 3 drinks on a given day for women and for men ≥ 65, and more than two drinks daily, more than 14 drinks weekly, or exceeding 4 drinks on a given day for men < 65 [8]. Pertinent to these guidelines, a standard drink is defined as a volume of beverage containing 14 grams of alcohol. This corresponds to a 12-oz (355 ml) beer containing 5% alcohol by volume, 5 oz. (148 ml) of wine containing 12% alcohol, or 1.5 oz. (44 ml) of spirits containing 40% alcohol. While modestly exceeding these limits in a sustained manner is unlikely to cause traditionally alcohol-related harms, this pattern is variably associated with increased morbidity in the long term relative to lighter drinking [9-11]. At the higher end of severity is the daily or frequent consumption of large amounts of alcohol, with sharply increased risks for alcohol dependence, cirrhosis, upper airway and gastrointestinal tract cancers, dementia, and other highly morbid conditions.

The prevalence of unhealthy use among hospital inpatients will depend on characteristics of hospitals and the populations they serve [12]. However, a systematic review of screening studies suggested an overall average of about 17% will self-report unhealthy use [13], with a 3-fold increase in men relative to women. This summary estimate is less than the prevalence in the general adult population [8]. However, because unhealthy alcohol use is most common in younger males, unhealthy use is felt to increase the risk for hospitalization after accounting for age and gender [14]. Among general hospital patients who do consume alcohol, regardless of the presence of unhealthy use, about one-quarter will have an alcohol use disorder, which is much higher relative to current drinkers in the general population [15-17]. In addition to their physical health problems, relative to the non-hospitalized alcohol dependent population, hospitalized alcohol-dependent individuals have a higher prevalence of polysubstance use and mental health comorbidities [18]. Thus, while inpatient clinicians provide care for patients representing the broad spectrum of unhealthy alcohol use, there is a particularly high prevalence of complex alcohol dependent patients. Because brief intervention is most effective in patients with less severe unhealthy alcohol use, this has profound implications for the effectiveness of hospital-based SBIRT. Of note, despite these increased health risks, the evidence on severe unhealthy alcohol use and early hospital readmission is mixed [19,20], and may depend on concurrent abuse of other drugs.

### Detecting and categorizing the severity of unhealthy alcohol use

The process of detecting and categorizing the severity of unhealthy alcohol use focuses on two aspects of alcohol involvement. Firstly, the amount of alcohol consumed must be quantified, and this will determine the presence or absence of unhealthy use. Secondly, alcohol-related consequences should be assessed to determine the presence of an alcohol use disorder.

### Assessment for unhealthy alcohol use

Alcohol use is often recorded in a hospital admission history & physical by the frequency with which a certain amount of alcohol is consumed. With this method, alcohol use should be estimated in an unambiguous manner using the definition of a standard drink, and aids are available to assist clinicians and their patients in estimating alcohol consumption [8]. Rather than non-standardized questioning to characterize alcohol use, a number of short instruments have been evaluated [21], and their brevity should facilitate standardized screening in the hospital. Some of these tools are listed in Table 1, including the AUDIT-C [22-24], quantity-frequency items [24,25] , and heavy drinking day question [26,27], any of which should target past-year drinking. The estimates shown in the table were derived from primary care samples [24,27], and could differ to some extent in hospitalized patients. None of the instruments are perfectly specific, and positive results should trigger additional inquiry to characterize drinking and confirm unhealthy alcohol use.

**Table 1:** Selected findings on brief screening tools for unhealthy alcohol use

**Screening tool**	**Positive result**	**Sensitivity**	**Specificity**	**Study Populations**
		**(95% CI)**	**(95% CI)**	
AUDIT-C	≥ 4 (men)	86 (80-92) men	89 (85-93) men	Primary care [24]
(3 items)*	≥ 3 (women)	73 (66-79) women	91 (89-93) women	
Quantity-Frequency	Exceeding recommended	88 (83-94) men	84 (80-89) men	Primary care [24]
(3 items)*	drinking limits	68 (61-75) women	91 (89-93) women	
Heavy-drinking day	Any heavy days (≥ 4 drinks women, ≥ 5 drinks men)	83 (71-90) men	72 (61-81) men	Primary care [27]
past year (1 item)		81 (64-91) women	84 (76-89) women	

*The AUDIT-C and quantity-frequency items were identical, with different interpretations of a positive result.

An added complexity of assessing for unhealthy alcohol use in hospitalized patients is illness severity, which may prohibit obtaining information directly from the patient. In such circumstances the presence of unhealthy alcohol use can often be estimated from past medical records or surrogate reporters. When alcohol-related disease is suspected blood alcohol levels can be helpful if heavy drinking occurred in the past 8 to 12 hours (an average elimination is roughly one drink per hour). Gamma-glutamyltransferase also has some utility, but will be less specific for chronic heavy drinking in medical settings [28]. Carbohydrate-deficient transferrin, another biomarker for chronic heavy drinking, has been shown to predict adverse perioperative events [29], but is limited by modest sensitivity. In addition, results may not be available for several days, limiting use for decision-making in the hospital. Newer alcohol consumption biomarkers that require ethanol for their synthesis (e.g., urine ethyl glucuronide [30], blood phosphatidylethanol [31]) are a research focus and clearly have potential clinical utility [32-39]). In general however, pending the development of cost-effective, widely-available assays at the point of care, use of these newer biomarkers in risk-stratifying for alcohol problems at the time of hospitalization is not yet feasible in most locations.

#### Assessment for alcohol use disorders in patients with unhealthy use

In patients with unhealthy alcohol use, characterization of severity requires an assessment of recent alcohol-related problems, which will often satisfy the criteria for an alcohol use disorder in hospitalized patients [16]. A number of screening instruments for alcohol use disorder have been developed, and some are listed in Table 2[21,40].

**Table 2:** Selected findings on brief screening tools for alcohol use disorders

**Instrument**	**Positive result**	**Sensitivity**	**Specificity**	**Study populations**
AUDIT	≥ 8	Range	Range	Systematic review of primary care studies [21]
(10 items)*		61 to 96	85 to 96	
CAGE	≥ 2	Range	Range	Systematic review of primary care studies [21]
(4 items)		77 to 94	79 to 97	
RAPS4	≥ 1	93	87	Emergency department patients [40]
(4 items)				

*AUDIT includes the AUDIT-C and can be used to screen for unhealthy alcohol use. Similarly, screens in Table 1 can be combined with screens in Table 2 to jointly screen for unhealthy alcohol use and alcohol use disorders.

Importantly, as for any ordinal screening instruments, different cutoff scores will result in a tradeoff between sensitivity and specificity. In addition, relatively low cutoff scores on an instrument that assesses both the magnitude of drinking and alcohol-related consequences can be used to screen for unhealthy alcohol use and alcohol use disorders (e.g., as with the AUDIT [41]). Ultimately however, a diagnosis of an alcohol use disorder should be based on an assessment of established criteria.

The American Psychiatric Association (Diagnostic and Statistical Manual/DSM) and World Health Organization (International Classification of Disease/ICD) have developed criteria for a diagnosis of current alcohol dependence that substantially overlap, and require the presence of at least 3 of the diagnostic criteria during the past 12 months (or co-occurring more distantly for alcohol dependence in remission). The current DSM-IV criteria for dependence and abuse are listed in Table 3. Importantly, due to evidence that alcohol problems exist on a continuum rather than sharply defined categories, the upcoming revision of the DSM in 2013 (DSM-V) will controversially do away with the terms “abuse” and “dependence”, and will have only “alcohol use disorder” [42,43]. Individuals satisfying 2 or 3 of the 11 criteria will be classified as having “moderate” alcohol use disorder, and those meeting 4 or more criteria will be classified with “severe” alcohol use disorder. This DSM-V reclassification may cause some initial confusion, but the concept of a single disorder that varies in severity will likely be a welcome change for many hospital-based clinicians.

**Table 3:** American Psychiatric Association criteria for alcohol use disorders

**DSM-IV Alcohol dependence**	**DSM-IV Alcohol abuse**	**DSM-V Alcohol use disorder**
(≥ 3 of the following)	(≥ 1 of the following)	(≥ 2 of the following)
• Tolerance	*Continued drinking despite*….	• Tolerance
• Withdrawal		• Withdrawal
• Repeatedly exceeding intended limits	• Increased risk for physical harm	• Repeatedly exceeding intended limits
• Spending a lot of time drinking or recovering from alcohol effects	• Trouble in important relationships	• Spending a lot of time drinking or recovering from alcohol effects
• Failed attempts to cut down or abstain	• Failure to perform important roles	• Failed attempts to cut down or abstain
• Continued drinking despite physical or psychological problems	• Legal problems*	• Continued drinking despite physical or psychological problems
• Spending less time on important activities due to drinking		• Spending less time on important activities due to drinking
		• Increased risk for physical harm
		
		• Trouble in important relationships
		• Failure to perform important roles
		• Craving for alcohol*

*“Legal problems” will be dropped as an alcohol use disorder criterion, and “craving” will be added.

### Treatment for unhealthy alcohol use

#### Treatment for non-dependent unhealthy alcohol use

After initial screening and clinical assessment, patients with unhealthy alcohol use will have been categorized as having or not having an alcohol use disorder. In the latter, the clinical effort should be aimed at drinking reduction to levels that are unlikely to contribute to health problems. To accomplish this, the evidence, while mixed, generally favors a modest effect of brief interventions during hospitalization. A meta-analysis [44] of 4 randomized controlled trials (one in trauma patients did not exclude dependence, two excluded dependence, and one excluded patients with alcohol-related physical problems) concluded that brief intervention for unhealthy drinkers reduced alcohol consumption by an average of approximately 5 drinks/week 6 months post-intervention. Exclusion of the trauma-based trial due to unblinded outcome assessment reduced the magnitude and statistical significance of this estimate. Reported alcohol use at one year was synthesized for 4 other studies (3 with variable exclusion of patients with alcohol use disorders) and, although there was roughly a 2 drink per week reduction in the intervention groups, this was not statistically significant relative to controls. Importantly, while the evidence base for outcomes other than alcohol use is relatively under-developed, this meta-analysis found evidence that brief intervention was associated with decreased mortality at one year. In addition, a US study of trauma patients reported a 47% reduction in recurrent trauma over the 3 years following receipt of brief intervention [45].

Overall, the evidence tends to favor brief intervention for hospitalized patients with less severe unhealthy alcohol use. A number of different strategies have been studied, but brief intervention in the hospital should include at a minimum feedback about alcohol use (including associations with conditions the patient may have such as hypertension), advice to reduce consumption to safer levels, an explanation of why these limits are recommended, a non-confrontational inquiry to determine a patient’s interest in reducing their drinking, and determination of a plan to achieve drinking goals [46]. This can be delivered by a variety of health care personnel [44,47], which can lower the cost of brief intervention.

#### Acute Treatment for alcohol use disorders (typically accompanied by frequent heavy drinking)

The potential for acute complications renders the detection of alcohol use disorders and associated heavy drinking particularly important at the time of hospitalization. A number of informative reviews are available for guiding care [7,48-50], and only a brief summary is provided here (Table 4). In patients with prior severe withdrawal or unstable medical disease, clinicians may wish to institute preventive measures for acute alcohol withdrawal with fixed dose benzodiazepines (e.g., chlordiazepoxide 50 mg every 6 hours for 24 hours followed by 25 mg every 6 hours for 48 hours) [48]. Patients must still be monitored for over-sedation or inadequate dosing. Active withdrawal can be managed with symptom-triggered or scheduled benzodiazepine dosing, with the former resulting in decreased benzodiazepine use and shorter treatment duration [51]. Several potential regimens are listed in Table 5[49,51-53], and use of a shorter-acting benzodiazepine (e.g., lorazepam) will require a taper to prevent recurrent symptoms. Monitoring and dosing must be guided by a validated symptom scale such as the Clinical Institute Withdrawal Assessment for Alcohol-revised (CIWA-Ar) or somewhat shorter CIWA-AD [54-56] administered by trained personnel. Inappropriate selection of patients for symptom-triggered therapy has been reported, with the major reasons being inability to communicate (and thus assess all withdrawal symptoms), lack of heavy drinking in the days preceding hospitalization, and other causes of delirium [57]. Thus an accurate history is required and other causes of delirium must always be considered. For example, chronic heavy drinking suppresses the immune system [58,59], and clinicians must remain vigilant for infections such as pneumonia or sepsis. Other causes of delirium (e.g., hypoxia, stroke, effects of drugs other than alcohol, etc.) may also need to be ruled out depending on the clinical circumstances.

**Table 4:** Immediate issues in the care of chronic heavy drinkers admitted to the hospital

**Clinical issue**	**Treatment**
Assess risk for nutritional deficiency	• Thiamine supplementation.
	• Possibly folate and multivitamin supplement.
Assess hydration status and electrolytes (risk for hypocalcemia and hypomagnesemia with or without hypokalemia and hypophosphatemia)	• IV or oral fluids.
	• Oral or IV electrolyte replacement.
Risk for acute alcohol withdrawal	• Close observation with validated instrument or prophylactic benzodiazepine, particularly in those with previous withdrawals or history of severe withdrawal (delirium tremens or seizure).
	• Prophylaxis still requires close observation for over or under-sedation.
Active alcohol withdrawal	• Symptom-triggered or scheduled benzodiazepine.
	• Close observation with validated instrument with either symptom-triggered or scheduled dosing.
	• Alternate medication (e.g., phenobarbital) in rare event that benzodiazepine is unsuccessful at controlling agitation.
	• Possible beta blocker or clonidine for autonomic manifestations if benzodiazepine alone is insufficient.
	• Possible haloperidol if benzodiazepine alone is insufficient for delirium.
	• Consider other causes of delirium.

**Table 5:** Examples of symptom-triggered regimens for alcohol withdrawal*†

**Initial oral medication dose**	**Frequency**	**Medication half-life**
Diazepam 10 to 20 mg if CIWA-Ar ≥ 8 to 10	Repeat same dose hourly until	Long half-life may provide smoother withdrawal, but may accumulate in elderly or those with liver disease.
	CIWA-Ar < 10	
Chlordiazepoxide 50 mg if CIWA-Ar > 9	Repeat 50 mg hourly until CIWA-Ar < 10	Intermediate half-life may provide smoother withdrawal than lorazepam.
Lorazepam 2 to 4 mg if CIWA-Ar ≥ 8 to 10	Repeat same dose hourly until	Short half-life may increase withdrawal symptoms between doses. May be better tolerated in elderly and liver disease patients.
	CIWA-Ar < 10	

*Fixed dose regimens generally consist of the same dose administered every 6 hours for 24 hours followed by half the initial dose every 6 hours for 48 hours. Close monitoring is still critical as adjustments in dose, frequency, and length of taper depend on clinical response.

†Detailed descriptions are found in citations 48 and 53.

Initial treatment of severe withdrawal may require intravenous benzodiazepines (e.g., 2 to 4 mg of lorazepam or 5 to 10 mg of diazepam) and additional dosing as needed to control symptoms. Usually benzodiazepines alone are sufficient, and a strong evidence base supports their use [60]. The rare patient with severe withdrawal who fails benzodiazepine treatment will require intensive monitoring and treatment with barbiturates or propofol [49]. Phenobarbital is most frequently used in this circumstance, with 30 mg having roughly equivalent effects on withdrawal symptoms as 2 mg lorazepam, 25 mg chlordiazepoxide, or 10 mg diazepam [53]. Some centers with expertise in alcohol withdrawal treatment use anticonvulsants (e.g., carbamazepine, gabapentin) as a primary prophylaxis or treatment rather than benzodiazepines, but the evidence base for justifying clinical recommendations is currently under-developed. In particular, anticonvulsants have not been shown to reduce the incidence of alcohol withdrawal seizure or delirium tremens [61]. The use of intravenous alcohol infusions continues in some locations [62], mainly in surgical specialties, due to some surgeons’ familiarity and comfort with this treatment. This approach has been discouraged due to potential toxicity (remembering that first-pass metabolism can be substantial in chronic heavy drinkers and is bypassed by this route), and evidence shows that this approach is at best no more efficacious for prophylaxis than benzodiazepines [63], and may have a high failure rate [64,65].

#### Pre-operative assessment for patients with an alcohol use disorder

Chronic heavy drinking (e.g., averaging at least 50 to 60 grams of alcohol per day or usually exceeding daily limits) increases perioperative morbidity due to increased risks for acute withdrawal syndrome, pneumonia and ARDS, wound infections, bleeding, myocardial dysfunction, and enhanced stress responses [59,66]. Thus pre-operative detection should trigger efforts at detoxification and achieving several weeks of abstinence before elective procedures. For the more likely emergent procedures in hospitalized patients, parenteral thiamine, electrolyte replacement, and close monitoring and treatment for post-operative withdrawal are important components of care, and causes of delirium other than withdrawal should be considered as they would be for patients who are not heavy drinkers. Prevention of acute withdrawal with benzodiazepines is an important consideration in this population [67], and perioperative morphine may reduce post-operative pneumonia by ameliorating neuroendocrine and immune imbalances induced by chronic heavy drinking and peri-operative abstinence [68]. Much of the perioperative management will naturally be directed by the anesthesiologist and surgeon, and any consulting physicians should make certain that these providers are aware of the patient’s heavy drinking. Of note, inappropriate use of symptom-triggered therapy for withdrawal has been observed in surgical patients [57]. It is equally important to communicate to all providers when a known heavy drinker has passed the time when initial manifestations of acute alcohol withdrawal would be expected (within 2 to 3 days of the last drink).

#### Additional pre-discharge treatment considerations for patients with an alcohol use disorder

Some research suggests hospitalization has the potential to facilitate treatment for alcohol use disorders by increasing recognition of the need to reduce drinking and intent to do so [69-72]. Despite this, brief interventions are unlikely to be effective at reducing drinking for hospitalized patients with alcohol dependence [73], but factors including avoidance of heavily drinking friends and engaging in alcohol treatment after hospitalization predict drinking reduction [71]. Thus referral for outpatient treatment should be pursued and integrated into brief intervention (i.e., SBIRT), but, even among research participants, overall compliance with referral is modest [73,74]. Brief intervention may be more effective in enhancing referral for women and younger adults [75]. Some evidence suggests that peer-involvement from volunteer Alcoholics Anonymous members as a supplement to brief intervention can increase treatment initiation and abstinence following hospitalization [76].

Following hospital discharge, integrated outpatient treatment (i.e., post-discharge care for alcoholism and medical conditions with the same healthcare provider) may be effective under ideal conditions [77,78]. A related approach involves a chronic disease management strategy in primary care with as-needed addiction specialist support, which may improve the quality of outpatient care and facilitate recovery [79,80]. Thus it is reasonable to refer patients to this mode of primary care if it exists locally.

Other potential interventions prior to discharge must be extrapolated from studies in treatment-seeking alcohol-dependent populations or treatment strategies implemented for other chronic medical and psychiatric conditions. Taking advantage of findings in the alcoholism pharmacotherapy field that show modest efficacy for several medications coupled with brief counseling strategies [81,82], an approach for alcohol dependent inpatients might include brief interventions and initiation of pharmacotherapy during the hospitalization. Several potential medications are listed in Table 6, together with a selection of their generally small effect sizes [83-87]. With the exception of disulfiram, medications for relapse prevention target brain receptors thought to be involved in relapse, including subsets of opioidergic, serotenergic, glutamatergic, and dopaminergic receptors [88], and complete abstinence may not be realized. As in the treatment of other conditions such as hypertension, reduced post-hospitalization drinking due to medication use may be considered a successful albeit partial response, even if the ultimate goal is abstinence. However, with the possible exception of transitional clinics following hospital discharge, continuation of medication will mainly be in the hands of ambulatory clinicians.

**Table 6:** Select Effects of Medications on Drinking Outcomes

**Medication**	**Drinking Outcome**	**Effect estimate**	**Source**	**Factors Influencing Medication Choice**
		**(95% CI or p-value)**		
Naltrexone	Heavy drinking day (≥ 60 grams alcohol)	Relative risk 0.83	Meta-analysis of 50	Avoid in patients with opioid abuse or use; caution in liver disease and advanced kidney disease
		(0.76-0.90)	randomized controlled trial (RCT’s) [87]	
Acamprosate	Any drinking	Relative risk 0.86	Meta-analysis of 24	Avoid with advanced kidney disease (e.g., creatinine clearance < 30 ml/min)
		(0.81-0.91)	RCT’s [86]	
Disulfiram	Any drinking	Slight majority of trials found improved abstinence.	Review of 11 RCT’s [85]	Avoid if alcohol-disulfiram reaction medically dangerous; number of medical conditions associated with accidental reaction; avoidance of alcohol-containing products
Topiramate*	% heavy drinking days	8.4% reduction	Multicenter RCT [83]	Caution with advanced liver or kidney disease; risk for metabolic acidosis with predisposing conditions; avoid abrupt discontinuation
		(3.1-13.8)		
Ondansetron*	Average number of drinks on days alcohol was consumed	*If alcoholism onset before age 25,* 4.28 relative to 6.9 in placebo(p=0.004)	RCT [84]	Not shown to be beneficial for later-onset alcohol dependence; may prolong QT interval

*Not FDA-approved for treating alcohol dependence.

### Unhealthy alcohol use and the joint commission

An impetus for a standardized approach to SBIRT for unhealthy alcohol use, is the recent inclusion by JCAHO of SBIRT as a quality care measure for hospitalized patients [5], and these recommendations have been summarized [6]. In brief, JCAHO has recommended screening for unhealthy use in all patients, brief intervention for patients with unhealthy use, in-hospital treatment or referral for alcohol use disorders, and consideration of medications for alcohol dependence treatment. Performance of these tasks would be measured by self-reported outcomes through phone contact within 2 weeks of discharge.

This JCAHO initiative is grounded in the recognition that unhealthy alcohol use and other drug use is a major determinant of health in hospitalized patients, but has been criticized due to insufficient evidence on the overall effectiveness of SBIRT among general hospital inpatients [6]. In that regard, the JCAHO quality initiative extends beyond the evidence, but represents an attempt to integrate research findings into medical care. This intent must be applauded, and should be used as a springboard to further develop methods that will improve patient outcomes following an index hospitalization. The more severe nature of unhealthy alcohol use in the hospital, the process of SBIRT, and the intended outcomes should drive the research agenda. Some thoughts in this regard are listed in Table 7, which is not meant to be exhaustive. Research efforts must be geared toward generic processes of care and quality assessment, as the intent will be to improve patient outcomes in settings where personnel with unique interests in this field are not always present.

**Table 7:** Potential research topics relevant to patient care and JCAHO quality measures

**Topic**	**Potential research foci**
Screening and assessment	•Is there reason to use more than a single heavy drinking day question to screen for unhealthy alcohol use?
	•Is there a better strategy than screening all admissions?
	•How should screening be integrated with electronic work flows?
	•What is the role of newer alcohol consumption biomarkers?
	•What is the optimal assessment method in the hospital?
	•What training will hospital-based clinicians require to enhance their skills and confidence in diagnosing alcohol use disorders?
	•How do patients feel about assessment during hospitalization?
Treatment	•What patients are most likely to respond to brief intervention?
	•How can the beneficial effects of brief intervention on alcohol use be increased?
	•How do we enhance the success of referral?
	•Does pharmacotherapy for relapse prevention work in this population?
	•What is the role for joint detection and treatment of other drug and mental health co-morbidities?
	•Can computerized support enhance treatment?
Measuring performance	•What are the effects of brief intervention on other outcomes such as progression of alcohol problems and hospital readmission?
	•What are the most pertinent patient-centered outcomes?
	•What is the optimal method for assessing the quality of hospital-based SBIRT?

### Implementation barriers and facilitators

In general, major barriers to incorporation of evidence-based care include organizational leadership, work capacity, training, ongoing support, and others [89], and SBIRT is no exception. A systematic review of qualitative data from 47 studies [90] identified major SBIRT-implementation barriers to be limited resources, training, support of management, and workload. Most results were from primary-care-based studies, but, consistent with these findings, critical components of implementing and sustaining SBIRT at a rural hospital in Australia included the support of hospital management and a dedicated project worker [91]. Similar issues were also identified for nursing-delivered SBIRT, with the addition of concerns over limited interdisciplinary collaboration about alcohol problems, compatibility with the acute care nursing role, lack of privacy, and concerns about patient attitudes [92]. This latter issue was also identified in a previous hospital-based study on SBIRT [93]. Regarding training needs, SBIRT training programs have been shown to increase provider comfort in assessing alcohol problems and have increased utilization of SBIRT [94], and on-line instructional programs are available to enhance dissemination (e.g., http://medicine.yale.edu/sbirt/index.aspx; http://www.bu.edu/bniart/sbirt-in-health-care/sbirt-educational-materials/sbirt-videos/). An additional barrier is the lack of an unambiguous tool for monitoring the quality of SBIRT, which ideally should include assessment of core brief intervention components rather than non-specific provider or patient report of alcohol counseling [6,95]. Interestingly, implementation of a performance measure and electronic reminders were each associated with an increase in the receipt of brief intervention in outpatient VA settings [96], and this type of strategy has the potential to enhance SBIRT performance in the hospital [97].

## Conclusions

Unhealthy alcohol use is common in hospitalized patients, with a high prevalence of severe alcohol problems among those with unhealthy use. Detection should include the use of a validated screening instrument to determine the presence of unhealthy use, and assessment of alcohol-related consequences in patients with positive screening results to categorize the severity of unhealthy use. Based on current evidence, patients without an alcohol use disorder should receive a brief intervention to target reduced drinking. Acute care issues for patients with alcohol use disorders have been well described and are standards of care. Additional research is needed to guide discharge planning for inpatients with an alcohol use disorder, but management should include referral to outpatient addiction treatment if available, consideration of medications to prevent a return to heavy drinking, and explicit follow-up on alcohol use in the ambulatory medical setting. JCAHO has advanced SBIRT for unhealthy alcohol use as a quality measure, and barriers to implementation are mainly generic factors rather than specific to SBIRT. Continued research is needed across the spectrum of unhealthy alcohol use to further demonstrate the benefits of hospital-based SBIRT, refine the process, and improve care for hospitalized patients.

## Competing interests

The authors declare that they have no competing interests.

## Authors’ contributions

RM and SHS each reviewed the literature and participated in drafting the manuscript. Both authors read and approved the final manuscript.
